# Parent-Adolescent Discrepancies in Perceiving Parental Psychological Control and Autonomy Support Predict Adolescents’ Psychological Adjustment: Does Adolescent Gender Make a Difference?

**DOI:** 10.1007/s10964-025-02144-5

**Published:** 2025-02-01

**Authors:** Jiayin Zheng, Bin-Bin Chen

**Affiliations:** 1 Faculty of Education, University of Cambridge, 184 Hills Rd., Cambridge, CB2 8PQ United Kingdom; 2 Department of Psychology, Fudan University, 220 Handan Rd., Shanghai, 200433 China

**Keywords:** Parent-adolescent discrepancy, Parent psychological control, Parent autonomy support, Psychological adjustment, Gender difference, Latent difference scores

## Abstract

Few studies simultaneously examined how parent-adolescent discrepancies in reporting psychological control and autonomy support predicted adolescents’ adjustment and the moderation by adolescent gender remains unknown. This longitudinal study addressed these gaps using a Chinese sample of 310 adolescents (158 girls; *M*_age_ = 13.34, *SD* = 0.36) and their parents. Adolescents reported depression and resilience and dyads reported parenting. The latent difference scores analysis showed higher psychological control and lower autonomy support perceived by adolescents than parents and larger parent-boy discrepancies in psychological control. Psychological control discrepancies predicted higher adolescents’ depression and autonomy support discrepancies predicted lower boys’ depression. The results suggest that parent-adolescent discrepant perceptions of different parenting behaviors predict adolescents’ adjustment via different processes, which vary for boys and girls.

## Introduction

Adolescence is a period when the need for self-reliance, independent decision-making, and volitional functioning is enhanced (Soenens et al., 2017). Whether adolescents’ need for autonomy is satisfied or frustrated by parenting behaviors such as psychological control and autonomy support can affect their psychological adjustment and well-being (Costa et al., 2019). The multi-informant assessment is a recommended practice to assess parenting behaviors for it integrates information from both parents’ and adolescents’ perspectives across different situations, thus avoiding single-informant biases (e.g., De Los Reyes & Ohannessian, 2016). Nonetheless, the cross-informant discrepancy in perceiving parenting behaviors has been discovered repeatedly (for a meta-analysis, see Hou et al., 2020), which has been suggested to serve as an independent variable that predicts a variety of outcomes regarding family functioning and adolescents’ development (Rescorla, 2016). Parents generally hold more positive views toward their parenting, reporting lower levels of psychological control and higher levels of autonomy support than their adolescents (Korelitz & Garber, 2016). However, few studies have examined parent-adolescent divergent perceptions of psychological control and autonomy support simultaneously within one sample and how those discrepancies affect adolescents’ psychological adjustment. Moreover, given the well-established understanding of gendered parenting behaviors (Morawska, 2020), little is known about whether parent-adolescent discrepancies in perceiving parenting differ between boys and girls. The current study aimed to examine the differences in parents’ and adolescents’ perceptions of psychological control and autonomy support, unpacking the potentially different processes through which the divergent perceptions of these two distinct parenting behaviors predicted adolescents’ depressive symptoms and resilience. Furthermore, the current study aimed to test the moderating effect of adolescent gender on the cross-informant discrepancies and the relations to adolescents’ outcomes, which was distinguished from previous studies focusing on the role of parent gender only.

### Parent-Adolescent Discrepancies in Perceiving Psychological Control and Autonomy Support

According to the self-determination theory (SDT)’s dual process model (Deci & Ryan, 2012), parental psychological control and autonomy support are two opposite types of parenting. Psychological control frustrates one’s need for autonomy by making children comply with parents’ own expectations and changing children’s thinking and behavioral patterns (Soenens & Vansteenkiste, 2010). By contrast, autonomy-supportive parenting encourages children’s self-initiation and provides children with a pertinent rationale when introducing rules (Ryan et al., 2015). Recent years have witnessed a growing use of multi-informant assessment of parenting to replace the single-informant assessment that leverages either parents’ self-reports or adolescents’ reports (De Los Reyes & Ohannessian, 2016). Counterintuitively, although family members such as parent-adolescent dyads live together, their understandings of family functioning are only lowly-to-moderately correlated (e.g., Korelitz & Garber, 2016). Studies using multi-informant assessment have demonstrated parents’ and adolescents’ divergent perceptions of parental psychological control and autonomy support: regardless of sociocultural contexts, adolescents reported higher psychological control than parents (e.g., Ingoglia et al., 2021); although less examined, autonomy support was rated lower by adolescents than by parents (e.g., Vrolijk et al., 2023). These studies suggested that the patterns of parent-adolescent discrepancies differed by the type of parenting behaviors. However, previous studies focused on either psychological control or autonomy support. Rarely have they tapped into the discrepancies in perceiving these two parenting behaviors simultaneously within one sample (for two exceptions, see Leung & Shek, 2014; Van Heel et al., 2019). Moreover, parenting behaviors such as psychological control perceived by adolescents may differ by adolescent gender (e.g., Luebbe et al., 2014). However, little is known whether parent-adolescent discrepancies in perceiving parenting differ between boys and girls.

### Parent-Adolescent Discrepancies and Adolescents’ Psychological Adjustment

Both parents’ and adolescents’ reports of parental psychological control and autonomy support have been suggested to be associated with adolescents’ psychological adjustment. A recent meta-analysis synthesizing the findings from 238 articles demonstrated that psychological control and autonomy support respectively made unique contributions to children’s ill-being (e.g., negative affect, depression) (*r* = 0.20, 95% CI [0.17, 0.23]) and well-being (e.g., life satisfaction, positive affect) (*r* = 0.26, 95% CI [0.23, 0.28]) after controlling each other’s effect (Bradshaw et al., 2024). In the recent decade, multi-informant research has further revealed that parent-adolescent discrepancies in perceiving these two parenting behaviors can predict certain developmental outcomes of adolescents as well, which provides information in addition to single informants’ perceptions (Rescorla, 2016). The Operations Triad Model has been used to account for the essentiality of informant discrepancies in perceiving family functioning to children (De Los Reyes & Ohannessian, 2016). This model suggests that divergent parent-child perceptions may result from children’s increasing desire for autonomy and their will to enhance autonomy, which contributes to positive developmental outcomes (Zhang & Wang, 2024). Or, parent-adolescent discrepancies can indicate maladaptive family processes such as parent-child miscommunication, which lead to negative outcomes (Fan et al., 2023).

A few studies have investigated the effects of parent-adolescent discrepancies in perceiving psychological control and autonomy support on adolescents’ psychological adjustment (e.g., Zhai et al., 2024). For instance, one study drawing on a sample of Chinese father-adolescent dyads and mother-adolescent dyads unveiled that adolescents reported higher psychological control than their parents (Zhai et al., 2024). Moreover, father-adolescent discrepancies were positively associated with adolescents’ anxiety concurrently and greater parent-adolescent incongruence predicted higher adolescents’ anxiety one year later. Another study leveraging a six-wave dataset discovered that parents on average reported higher levels of autonomy support than adolescents (Vrolijk et al., 2023). The results of the random-intercept cross-lagged model in this study demonstrated positive concurrent associations between parent-child discrepancies and parents’ and adolescents’ depression, but no longitudinal associations. However, neither of the two studies examined parent-adolescent discrepancies in perceiving psychological control and autonomy support simultaneously within one sample, which may or may not predict adolescent outcomes via the same process. Moreover, they focused on adolescents’ psychological adjustment difficulties (i.e., anxiety and depression). The evidence for the effect of parent-adolescent divergent perceptions on adolescents’ positive outcomes is lacking.

Furthermore, evidence suggests that boys and girls may be differentially impacted by parenting. For instance, the diathesis-stress model suggests that boys may be more vulnerable to parental negativity such as parental intrusiveness and lack of parental sensitivity than girls (e.g., Barnett & Scaramella, 2013). The differential susceptibility model, however, states that boys may be not only more vulnerable to negativity but also more sensitive to positivity such as parental responsiveness than girls (e.g., Bacikova-Sleskova et al., 2024). However, the moderating effect of adolescent gender on parent-adolescent discrepancies in perceiving parenting behaviors remains unclear from the literature. Neither have previous studies investigated whether the effects of parent-adolescent discrepancies on psychological adjustment differ as a function of adolescent gender.

## The Current Study

Few studies have comprehensively examined the effects of parent-adolescent discrepant perceptions of psychological control and autonomy support on the positive and negative aspects of adolescents’ adjustment. Neither is known whether parent-adolescent discrepancy or its effects differ between parent-boy and parent-girl dyads. The current study aimed to examine parent-adolescent discrepancies in perceiving psychological control and autonomy support and how these discrepancies predicted adolescents’ depressive symptoms and resilience one year later via a relatively innovative analytical approach, i.e., the latent difference score modeling. Furthermore, the current study aimed to investigate the moderating effect of adolescent gender on the discrepancies and their associations with adolescents’ psychological adjustment. It was hypothesized that adolescents perceived higher psychological control and lower autonomy support than parents (Hypothesis 1). It was expected to find that parent-adolescent discrepancies were associated with adolescents’ depression and resilience (Hypothesis 2). Lastly, the moderating effect of adolescent gender on the parent-adolescent discrepancies and the relations of discrepancies to adolescents’ psychological adjustment was expected (Hypothesis 3). For the scarcity of research on these research questions, a relatively exploratory perspective was taken while making the hypotheses.

## Methods

### Participants

The current sample of 310 Chinese parent-adolescent dyads was recruited from three middle schools mainly enrolling students from middle-class backgrounds in Shanghai, China. Adolescents were in Grade 7 (158 girls; *M*_age_ = 13.34 years, *SD* = 0.36) at Time 1. Participating parents were adolescents’ primary caregivers (67% mothers; *M*_age_ = 41.64 years, *SD* = 3.84). Among them, 49% of the fathers and 42% of the mothers completed education beyond high school (e.g., a bachelor’s degree or higher). Given the current research context in an economically developed city, the participating parents on average received longer years of schooling (*M* = 14.16 years) than the national labor population (aged between 20–59 years old) in China (*M* = 11.7 years) at the time of data collection (National Bureau of Statistics of China, 2021).

### Procedure

This longitudinal dataset was gathered from parent-adolescent dyads through digitalized questionnaires at two time points spanning one year of time. Among the 418 parent-adolescent dyads participating at Time 1, 310 of them were retained in the study at Time 2. The high attrition rate was mainly due to the Covid-19 disruptions to schools. The dyads that completed two waves of data collection (*N* = 310) were included in the current analyses. The non-retained participants did not significantly differ from study completers in adolescent age and gender, *F*s ≤ 1.00, *p*s > 0.05, but parents were marginally younger for study completers than the non-retained, *F* = 3.74, *p* = 0.054, and parental education for study completers was lower than the non-retained, paternal education: *F* = 19.49, *p* < 0.001; maternal education: *F* = 19.18, *p* < 0.001. At each time point, participants were given detailed explanations of the research and completed the digitalized consent form before they started to respond to the questionnaire. The ethics review committee in the School of Social Development and Public Policy at Fudan University reviewed and approved all the study procedures and materials. Gifts were given to participating families as compensation for their time.

### Measures

#### Psychological Control

At Time 1, both adolescents and parents respectively completed a 10-item measure to report parental psychological control, which was a reduced form of an originally 18-item questionnaire (Wang et al., 2007). Adolescents reported how often their parents adopted psychological control in their parenting (e.g., “My parents tell me that I should feel guilty when I do not meet their expectations”) on a 5-point Likert scale (1 = *never*, 5 = *very often*). Parents completed the parallel items which were rephrased to the parental perspective. The one-factor latent structure was established respectively for parent and child measures through confirmatory factor analysis (CFA). Item 9 (“For things in my child’s life, I’m usually in charge”) was removed due to a low factor loading (<0.60; MacCallum et al., 1999). Adequate model fit was achieved by allowing the residual of a few items to co-vary (for details of CFA, see Table S2). Cronbach’s *α* = 0.94 for both adolescents’ and parents’ reports suggested high internal consistency.

#### Autonomy Support

At Time 1, both parents and children respectively completed an eight-item measure to report parental autonomy support, which was adapted from previous research (Wang et al., 2007). Adolescents reported on how often their parents provided autonomy support in their parenting (e.g., “My parents allow me to make choices for myself whenever possible”) on a 5-point Likert scale (1 = *never*, 5 = *very often*). Parents completed the parallel items which were rephrased to the parental perspective. The one-factor latent structure was established respectively for parent and child measures through CFA. Adequate model fit was achieved by allowing the residual of a few items to co-vary (for details of CFA, see Table S2 in the Online Resource). Cronbach’s *α* = 0.93 for adolescents’ reports and Cronbach’s *α* = 0.95 for parents’ reports suggested high internal consistency.

#### Adolescents’ Psychological Adjustment

##### Depressive Symptoms

At both Time 1 and Time 2, adolescents reported their depressive symptoms using the 12-item Short Mood and Feelings Questionnaire (SMFQ; Angold et al., 1995). Adolescents rated how often they felt depressed in the past two weeks (e.g., “felt miserable or unhappy”) on a 5-point Likert scale (1 = *never*, 5 = *very often*). All the items were averaged so that higher scores indicated greater depression. Cronbach’s *α* = 0.95 at Time 1 and Cronbach’s *α* = 0.98 at Time 2 indicated high internal consistency.

##### Resilience

At Time 1 and Time 2, adolescents rated their ability to bounce back from frustration and adversity using the 6-item Brief Resilience scale (BRS; Smith et al., 2008). Adolescents reported the degree to which they agreed with each statement (e.g., “I tend to bounce back quickly after hard times”) on a 5-point Likert scale (1 = *strongly disagree*, 5 = *strongly agree*). The mean was taken across all the items (three negatively worded items were reversed) so that higher scores indicated stronger resilience. Cronbach’s *α* = 0.78 at Time 1 and Cronbach’s *α* = 0.63 at Time 2 indicated acceptable internal consistency.

### Analytical Strategies

The preliminary analyses were conducted using IBM SPSS Statistics (Version 28) and the main analyses were conducted using *Mplus* Version 8 (Muthén & Muthén, 1998). The latent difference score (LDS) modeling was adopted to examine the parent-child discrepancies in perceiving parental psychological control and autonomy support and how the discrepancies predicted adolescents’ psychological adjustment. LDS is a flexible approach to examining informant discrepancies, which uses second-order latent factors to examine informants’ different perceptions of the same behaviors (De Haan et al., 2018). Compared to the traditional approaches such as Observed Difference Scores, LDS constructs one latent factor representing the convergent part of two informants’ reports and one latent factor representing the unique part of one of the informants’ report (i.e., the discrepancy score). Thereby, LDS empirically weights the effects of these two factors on other variables without confounding these two effects (De Haan et al., 2018). Compared to Polynomial Regression Analysis, LDS takes measurement errors into consideration and establishes measurement invariance across informants, leveraging the strengths of the structural equation modeling analysis (Murray et al., 2022).

As the first step, the measurement invariance of psychological control and autonomy support across parents and adolescents was tested using a series of multi-group confirmatory factor analyses. In all models, residual variances of parallel items across informants were allowed to co-vary (De Haan et al., 2018). The measurement invariance was assessed by comparing increasingly stringent models from the configural to the scalar invariance model. The measurement invariance criteria in Chen (2007) instead of the strict χ^2^ difference testing approach were used to identify a proper degree of invariance between informants without rejecting models with only minor non-invariance (e.g., Zhang & Wang, 2024). Measurement invariance was regarded as achieved if ΔCFI < 0.010, ΔRMSEA < 0.015, and ΔSRMR < 0.030 (metric invariance relative to configural invariance) or ΔSRMR < 0.010 (scalar invariance relative to metric invariance). After measurement invariance was achieved, the second step was to fit an LDS model respectively for parental psychological control and autonomy support (De Haan et al., 2018). First, latent factors representing the parents’ self-reports and adolescents’ reports were constructed from the observed items. Then, parent-adolescent discrepancies were modeled as a second-order latent factor in the model as the following equation:

$${{\rm{Y}}}_{{\rm{other}}{\hbox{-}}{\rm{report}}}=1{\ast} {{\rm{Y}}}_{{\rm{self}}{\hbox{-}}{\rm{report}}}+1{\ast} {\Delta }_{{\rm{self}},{\rm{other}}}$$

**Fig. 1:**
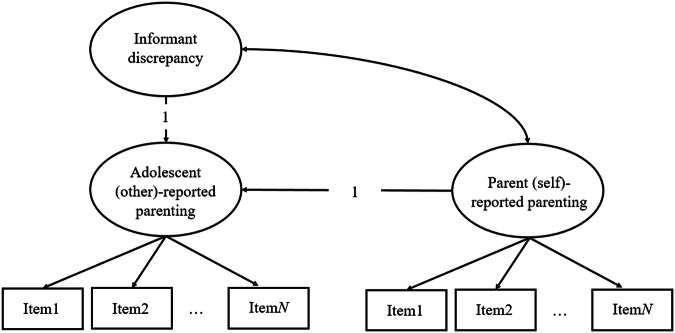
A Simplified Diagram for the Basic LDS Model to Estimate the Discrepancy between Parent Self-Reported and Adolescent-Reported Psychological Control / Autonomy Support. *Note*. By constraining the factor loadings of parent-report and informant discrepancy to be equal to 1, the results of a subtraction can be simulated. The resulting informant discrepancy score is the proportion of the adolescent-report that is not identical to parent-report

According to a simplified diagram in Fig. 1, LDS is the portion of the other-reported score that is not identical to the self-reported score, which is the subtraction of parents’ self-reports from adolescents’ reports. LDS scores include latent mean, variance, and covariance. The positive mean indicates higher adolescents’ reports than parents’, and the negative mean indicates lower adolescents’ reports than parents’. The latent variance indicates the differences in the degree of cross-informant discrepancies in different parent-child dyads within a sample. The covariance indicates the degree to which cross-informant discrepancy is correlated with parents’ self-report. The multi-group analyses were further conducted to examine the moderating effect of adolescent gender on the latent difference mean. The latent difference mean was allowed to be freely estimated in both boy and girl groups in the unconstrained model and it was fixed to be equal in the constrained model. If the model constraint did not lead to a significant change in the χ^2^ value (*p* < 0.05), the latent difference mean was invariant across groups.

After the basic LDS model was constructed, the model for psychological control and autonomy support was respectively estimated to further examine the associations between parents’ self-reports, parent-adolescent discrepancies, and adolescents’ psychological adjustment. In both models, variables of adolescents’ psychological adjustment at Time 2 were regressed on the factors of parents’ self-report and the latent difference score of psychological control/autonomy support. The effect of adolescents’ psychological adjustment at Time 1 was controlled for by estimating autoregressive paths. Psychological adjustment variables at Time 1 were allowed to co-vary with the factors of parents’ self-report and the latent difference score. The effects of parent gender, adolescent gender, and parents’ years of education on the factors of parents’ self-report and the latent difference score were controlled for. The effects of parents’ years of education and adolescent gender on adolescents’ psychological adjustment were controlled for.

Multi-group analyses were further conducted to examine whether the associations between parents’ self-reports, parent-adolescent discrepancies, and adolescents’ psychological adjustment varied by adolescent gender. The multi-group analyses involved two steps. First, all the parameters were freely estimated between groups. Second, a series of models with parallel paths between groups being constrained to be invariant were estimated. From Model 1 to Model 6 (See Tables S3–S4 in the Online Resource), one pair of parallel paths were constrained to be equal each time and tested whether the model constraint caused a significant change in χ^2^ value (*p* < 0.05), which suggested a between-group difference. In the fully constrained model (Model 7), all the parallel paths between groups were constrained to be equal.

There were no missing values in all the key study variables (including scale-level and item-level data) since only the families completing two waves of data collection were included in the current study. The absolute values of skewness ≤0.90 and kurtosis ≤1.40 of all the key study variables suggested that the data were in general normally distributed (Kline, 2016). Acceptable model fit was evaluated according to the following criteria: Comparative Fit Index (CFI) ≥ 0.90, Tucker Lewis index (TLI) ≥ 0.90, Root Mean Square Error of Approximation (RMSEA) ≤ 0.08, and Standardized Root Mean Square Residual (SRMR) ≤ 0.08 (Kline, 2016).

## Results

### Preliminary Analysis

Table S1 in the Online Resource presents descriptive statistics and zero-order correlations between the raw scores of key study variables. Parent- and adolescent-reported psychological control, *r* = 0.26, *p* < 0.001, and autonomy support, *r* = 0.24, *p* < 0.001, were weakly correlated with each other. Adolescent-reported psychological control and autonomy support were weakly-to-moderately correlated with adolescents’ psychological adjustment (except for the non-significant correlation between psychological control and resilience at Time 2) at both time points in the expected directions, 0.17 ≤ |*r*|s ≤ 0.40, *p*s < 0.05. Parent-reported psychological control were weakly correlated with adolescents’ psychological adjustment at Time 1, 0.12 ≤ |*r*|s ≤ 0.16, *p*s < 0.05. Parent-reported autonomy support were weakly correlated with adolescents’ psychological adjustment (except for resilience at Time 1) at both time points in the expected directions, 0.12 ≤ |*r*|s ≤ 0.16, *p*s < 0.05.

### Measurement Invariance

The results of measurement invariance tests are presented in Table 1. Scalar invariance across parent- and adolescent-reported psychological control and autonomy support was not fully supported (Chen, 2007). It has been suggested that unbiased inferences on latent means and variances can be achieved as long as no less than two observed items per latent factor had equal intercepts across informants (Van De Schoot et al., 2012; Zhang & Wang, 2024). A recent simulation study demonstrated that even when up to 80% of the items were non-invariant, unbiased inferences were possible (Pokropek et al., 2019). Thus, the intercepts of some of the items for psychological control and autonomy support were allowed to be freely estimated between informants to achieve partial scalar invariance. Specifically, the intercepts of Item 1 (“I let my child know that what I want him/her to do is the best for him/her and he/she should not question it”) and Item 3 (“I tell my child that when he/she grows up, he/she will appreciate all the decisions I have made for him/her”) of psychological control were allowed to vary across informants. For autonomy support, except for Item 6 (“I let my child make his/her own plans for things he/she wants to do”) and Item 7 (“I encourage my child to give his/her ideas and opinions when it comes to decisions about him/her”), the intercepts of the rest items were allowed to vary across informants.

**Table 1:** Goodness of fit indices of the confirmatory factor analysis models with Configural (A), Metric (B), Scalar (C), and Partial Scalar (C′) measurement invariance across informants

	Model fit indices						Model comparisons						
	Model	χ^2^	df	CFI	RMSEA	SRMR		Δχ^2^	df	*p*	ΔCFI	ΔRMSEA	ΔSRMR
Psychological control	A. Configural	97.085	40	0.978	0.068	0.032							
	**B. Metric**	**116.255**	**48**	**0.973**	**0.068**	**0.042**	**B vs. A**	**19.170**	**8**	**0.015**	–**0.005**	**0.000**	**0.010**
	C. Scalar	168.868	56	0.956	0.081	0.057	C vs. B	52.610	8	<0.001	–0.170	0.013	0.015
	**C′. Partial Scalar**	**132.636**	**54**	**0.969**	**0.069**	**0.049**	**C′ vs B**	**16.381**	**6**	**<0.001**	–**0.004**	**0.001**	**0.007**
Autonomy support	A. Configural	102.130	36	0.966	0.077	0.029							
	**B. Metric**	**118.388**	**43**	**0.961**	**0.075**	**0.056**	**B vs. A**	**16.258**	**7**	**0.070**	–**0.005**	–**0.002**	**0.027**
	C. Scalar	163.160	50	0.942	0.085	0.080	C vs. B	44.772	7	<0.001	–0.019	0.010	0.024
	**C′. Partial Scalar**	**123.217**	**44**	**0.959**	**0.076**	**0.062**	**C′ vs. B**	**4.919**	**1**	**0.028**	–**0.002**	**0.001**	**0.006**

The models achieving measurement invariance according to the criteria by Chen (2007) are in bold

*CFI* comparative fit index. *RMSEA* Root mean square error of approximation, *SRMR* Standardized root mean square residual

### Parent-Adolescent Latent Difference Scores

Latent means and variances of the differences between parent- and adolescent-reported psychological control and autonomy support are presented in Table 2. Specifically, adolescents perceived higher psychological control than parents (*M* = 0.365, *p* < 0.001, 95% CI = [0.235, 0.495]). By contrast, adolescents perceived lower autonomy support than parents (*M* = –0.366, *p* < 0.001, 95% CI = [–0.503, –0.230]). Significant variances of parents’ self-reports and latent difference scores suggested that there were significant between-individual differences in parents’ perceptions of psychological control and autonomy support as well as in the degree to which parents and adolescents differed in their perceptions in the current sample. Parent self-reported psychological control was negatively correlated with latent difference mean, *r* = –0.42, *p* < 0.001, suggesting that the lower parents rated their use of psychological control, the more likely adolescents overrated it. Parent self-reported autonomy support was negatively correlated with the latent difference mean, *r* = –0.25, *p* < 0.001, suggesting that the lower parents rated their use of autonomy support, the more likely adolescents underrated it.

**Table 2:** Model fit indices, latent means, and variances of parent self-reports and parent-child Latent Difference Score (LDS) in the basic LDS model

		Model fit indices						Means		Variances	
		χ^2^	df	CFI	TLI	RMSEA	SRMR	Self-report	Discrepancy	Self-report	Discrepancy
Psychological control	Overall	227.434	125	0.969	0.962	0.051	0.053	0.000	0.365^***^	0.607^***^	1.139^***^
	Boy	421.929	269	0.955	0.949	0.061	0.071	0.000	0.556^***^	0.697^***^	1.228^***^
	Girl							0.000	0.204^*^	0.583^***^	1.128^***^
Autonomy support	Overall	211.218	99	0.959	0.951	0.060	0.054	0.000	–0.366^***^	0.419^***^	1.160^***^

Latent means of parents’ self-reported psychological control and autonomy support were set to zero for the model identification. For multi-group analyses, only the significant difference between parent-boy dyads and parent-girl dyads in perceiving psychological control is presented

*CFI* comparative fit index, *TLI* tucker lewis index, *RMSEA* root mean square error of approximation, *SRMR* standardized root mean square residual

^*^p < 0.05. ^***^p < 0.001

According to the multi-group analyses, for psychological control, constraining the latent difference mean to be equal between boy and girl groups caused significant χ^2^ value change (Δχ^2^ (1) = 9.59, *p* = 0.002), suggesting a gender difference. The latent difference mean was 0.556 for boys, and 0.204 for girls, demonstrating that parent-boy dyads had larger discrepancies compared to parent-girl dyads. For autonomy support, constraining the latent difference mean to be equal between groups did not cause significant χ^2^ value change (Δχ^2^ (1) = 0.74, *p* = 0.389), suggesting no significant gender difference.

### Parent-Adolescent Discrepancy and Adolescents’ Psychological Adjustment

Relations of parents’ self-reports and the latent difference means to adolescents’ psychological adjustment are presented in Table 3. The model for psychological control provided an adequate fit to the data, RMSEA = 0.045, 90% CI [0.036, 0.053], CFI = 0.963, TLI = 0.954, SRMR = 0.051. So did the model for autonomy support, RMSEA = 0.051, 90% CI [0.043, 0.060], CFI = 0.955, TLI = 0.945, SRMR = 0.048.

**Table 3:** Associations between Parents’ self-reports, parent-child difference scores, and Adolescents’ psychological adjustment

		Depression			Resilience		
		*B*	*SE*	*β*	*B*	*SE*	*β*
Parent self-report (psychological control)	Overall	0.23	0.10	0.17^*^	–0.08	0.06	–0.09
Latent difference score mean (psychological control)	Overall	0.20	0.08	0.20^**^	–0.06	0.05	–0.09
Parent self-report (autonomy support)	Overall	–0.34	0.10	–0.21^**^	0.15	0.06	0.14^*^
Latent difference score mean (autonomy support)	Overall	–0.22	0.07	–0.22^**^	0.06	0.04	0.09
Latent difference score mean (autonomy support)	Boy	–0.34	0.08	–0.37^***^			
	Girl	–0.07	0.09	–0.06			

Significant standardized coefficients are in bold. For multi-group analyses, only the path differing between boys and girls is presented

*B* unstandardized estimate, *β* standardized estimate, *SE* standard errors

^*^p < 0.05. ^**^p < 0.01. ^***^p < 0.001

A higher parent-adolescent discrepancy in reporting parental psychological control predicted more adolescent-reported depressive symptoms, *β* = 0.20, *p* = 0.007, 95% CI = [0.05, 0.35], whereas it was not significantly associated with adolescent-reported resilience at Time 2, *β* = –0.09, *p* = 0.179, 95% CI = [–0.23, 0.04]. This suggested that the adolescents who were more likely to overrate their parents’ psychological control than others would perceive higher depression one year later, while their resilience was not affected by this discrepancy. Meanwhile, higher parent-reported psychological control was positively associated with adolescents’ depression, *β* = 0.17, *p* = 0.014, 95% CI = [0.03, 0.30], but not with adolescents’ resilience, *β* = –0.09, *p* = 0.192, 95% CI = [–0.23, 0.05].

A higher parent-adolescent discrepancy in reporting parental autonomy support predicted less adolescent-reported depressive symptoms, *β* = –0.22, *p* = 0.002, 95% CI = [–0.36, –0.08], whereas it was not significantly associated with adolescent-reported resilience at Time 2, *β* = 0.09, *p* = 0.133, 95% CI = [–0.03, 0.22]. This suggested that the adolescents who were more likely to underrate their parents’ autonomy support than others would have lower levels of depression one year later, while their resilience was not affected by this discrepancy. Meanwhile, higher parent-reported autonomy support was negatively associated with adolescents’ depression, *β* = –0.21, *p* = 0.001, 95% CI = [–0.33, –0.09], and positively associated with adolescents’ resilience at Time 2, *β* = 0.14, *p* = 0.016, 95% CI = [0.03, 0.25].

### Moderating Effect of Adolescent Gender on the Associations

Multi-group analyses demonstrated no gender difference in the effects of parent-reported psychological control and parent-adolescent discrepancies on adolescents’ psychological adjustment. Adolescent gender moderated the effect of parent-adolescent discrepancies in perceiving autonomy support on adolescents’ depression: the latent difference means negatively predicted boys’ depression one year later, *β* = –0.37, *p* < 0.001, 95% CI = [–0.54, –0.20], whereas it was not related to girls’ depression, *β* = –0.06, *p* = 0.487, 95% CI = [–0.24, 0.11].

### Sensitivity Analyses

Sensitivity analyses were conducted to examine whether the same conclusions were held when adolescents’ depression and resilience were entered into the models separately. These analyses did not lead to significant changes to conclusions (for details, see Table S5 in the Online Resource). Specifically, parent-adolescent discrepancies in perceiving psychological control still positively predicted adolescents’ depression, *β* = 0.19, *p* = 0.014, 95% CI [0.04, 0.33], but not adolescents’ resilience. Parent-adolescent discrepancies in perceiving autonomy support still negatively predicted adolescents’ depression, *β* = –0.21, *p* = 0.003, 95% CI [–0.35, –0.07], but not adolescents’ resilience. Parent-adolescent discrepancies in perceiving autonomy support were negatively associated with boys’ depression, *β* = –0.37, *p* < 0.001, 95% CI [–0.54, –0.20], but not with girls’.

## Discussion

With an increased need for autonomy, adolescents perceive parenting behaviors differently from parents, which affects adolescents’ adjustment. The current study advanced prior research by combining the investigations of two distinct parenting behaviors (psychological control and autonomy support) and by unveiling the differences between parent-boy and parent-girl dyads. The results showed that Chinese adolescents perceived higher psychological control and lower autonomy support than their parents. A larger difference was found in parent-boy dyads’ perceptions of psychological control than in parent-girl dyads’. Moreover, psychological control discrepancies predicted higher levels of adolescents’ depression one year later, whereas autonomy support discrepancies predicted lower levels of depression. Parent-adolescent discrepancies could not predict adolescents’ resilience. Adolescent gender moderated the association between the difference score of autonomy support and depression: a negative association was found for boys, but not for girls.

### Parent-Adolescent Discrepancies in Perceiving Psychological Control and Autonomy Support

After confirming that the two parenting questionnaires measured the same constructs for parents and adolescents, the current study discovered that adolescents reported higher levels of parental psychological control and lower levels of autonomy support than parents, which was in line with previous research (e.g., Korelitz & Garber, 2016). These patterns can be accounted for by the developmental stake hypothesis which posits that parents and children hold different developmental stakes (Gao et al., 2022). This is particularly so during adolescence when individuals show a growing desire to pursue autonomy and minimize connections with parents, which conflicts with parents’ stake in maintaining closeness with their children (Gao et al., 2022). On the one hand, parents tended to underrate their use of psychological control because they legitimated it as a parenting strategy to take care of their children, whereas adolescents might over-rate parents’ psychological control when they felt deprived of autonomy (Zhai et al., 2024). On the other hand, adolescents had a desire for autonomy stronger than ever so they were likely to feel they were not granted adequate autonomy support from parents and provided underestimated ratings, whereas parents tended to consider they had provided enough autonomy support (Ingoglia et al., 2021). The pattern that parents’ reports were more favorable toward themselves could also be accounted for by social desirability which is common to parents’ self-report measures (Bornstein et al., 2015). Parents did not want to unveil their use of psychological control and tried to portray themselves as autonomy-supportive parents to meet the common social expectations (Ingoglia et al., 2021).

### Parent-Adolescent Discrepancies Predicting Adolescents’ Psychological Adjustment

The results suggested that parent-adolescent discrepancies predicted adolescents’ depressive symptoms one year later, but not their resilience. While most studies investigating cross-informant discrepancies adopted a cross-sectional design to examine the concurrent associations, the current study demonstrated that the parent-adolescent discrepancies were able to predict adolescent outcomes longitudinally. As the previous research on parent-child discrepancies, this finding provided support for the Operations Triad Model (De Los Reyes & Ohannessian, 2016). On the one hand, parent-child discrepancies in perceiving parenting to some extent reflect miscommunication and mismatched understandings between family members, which may lead to negative developmental outcomes. The positive association between psychological control discrepancies and adolescents’ depression complied with this explanation. On the other hand, the finding that the greater the disagreement in reporting autonomy support, the lower adolescents’ depression reflected another aspect of the Operations Triad Model: adolescents’ disagreement with parents in perceiving autonomy support mirrored adolescents’ recognition and emphasis on autonomy, which would benefit their psychological adjustment. However, it is worth noting that one previous study using a Dutch sample found no evidence for longitudinal associations between parent-adolescent discrepancies in perceiving parental autonomy support and parents’ and adolescents’ depression via a six-wave cross-lagged model (Vrolijk et al., 2023). Thus, although yielded in a different sociocultural context, the current finding of longitudinal effects is worth revisiting with more waves of data collected from Chinese samples.

The findings regarding adolescents’ resilience were not as hypothesized. No evidence was found for the associations between parent-adolescent discrepancies and adolescents’ resilience, indicating that either parent-adolescent miscommunication or adolescents’ aspiration for autonomy that possibly underlies parent-adolescent discrepancies could influence adolescents’ self-perceived resilience ability. This result was different from one previous study showing that parent-adolescent (dis)agreement in reporting on family functioning could predict adolescents’ self-efficacy, resilience, and cognitive competence (Leung et al., 2016). However, this study examined the effect of interaction between parents’ and adolescents’ reports using the polynomial regression analysis, which was a different analytical approach from the latent difference score modeling that reveals the degree of between-informant discrepancies (i.e., mean difference). The scanty research and the existing inconsistent findings suggested that the influence of parent-adolescent discrepancies on positive adolescent outcomes such as resilience deserves the same empirical attention as adolescents’ maladjustment in future investigations. It is noted that adolescents’ self-perceived resilience was predicted positively by parent-reported autonomy support in the current study, which suggested that resilience was not a fixed personality trait. Rather, resilience was to some extent dynamic and could be enhanced through positive parenting (Ding et al., 2023), but it was not affected by negative parenting.

### The Gender Effect on Parent-Adolescent Discrepancies

The current study tested the parent-adolescent discrepancies and their relations to adolescent outcomes conditional on adolescent gender. The differentiated effects of parenting behaviors on boys and girls have been found across cultures, which reflect not only parents’ adoption of differential parenting strategies but also boys’ and girls’ different levels of susceptibility to parenting behaviors (Morawska, 2020). In the current sample, on the one hand, boys had a larger divergence from parents’ perception of psychological control than girls, suggesting that boys might be more sensitive to parental negativity (e.g., Barnett & Scaramella, 2013). This finding was in line with the diathesis-stress model which posits boys’ potentially higher vulnerability to negative parenting than girls. Nonetheless, the associations between psychological control discrepancies and adolescent outcomes were not different between boys and girls, indicating that this gender difference might be moderated by some unmeasured variables such as peer relationships, self-esteem, and stressful life events that could influence adolescents’ depressive symptoms (e.g., Wang et al., 2023). On the other hand, although we did not find the gender difference in the parent-adolescent discrepancies in reporting autonomy support, we found that boys’ rather than girls’ depression could be negatively predicted by autonomy support discrepancies. This might indicate that in the current Chinese sample, boys were cultivated to be more willing to fight for autonomy or even seek others’ support when they became aware of their deficiency in autonomy and unsatisfied with their parents’ autonomy granting (Zhang & Wang, 2024), which might boost their psychological well-being accordingly. Whereas certain stereotypically feminine traits such as being compliant and interdependent were expected from girls (e.g., Proudfoot & Kay, 2023). So even when girls did not receive adequate parental autonomy support as they wished for, they would not try to pursue it. However, more empirical support for this explanation is needed. The gender difference in the parent-adolescent discrepancies and the influence on adolescents’ psychological adjustment requires further investigation.

### Limitations and Future Directions

Although the current study made novel investigations of parent-adolescent discrepancies in perceiving parenting behaviors and their relations to later adolescents’ psychological adjustment using an advanced analytical approach and preliminarily tested the moderating effect of adolescent gender, several limitations deserve noting. First, it is worth mentioning that as in previous investigations (e.g., Murray et al., 2022; Zhang & Wang, 2024), only partial invariance between parents’ and adolescents’ reports was achieved for the parenting measures used in the current study. Ideally, future studies should pursue a full invariance to more solidly compute the latent between-informant discrepancies. Second, the self-report measure of adolescents’ resilience used in the current study might not reflect the nature of resilience as a dynamic process, which deserves reexamination using the measures such as dense repeated measures and time series that actually capture the resilience process (e.g., Hill et al., 2024). Third, the current study was not designed to have parallel father-adolescent and mother-adolescent dyads to enable a comprehensive examination within the family systems. Previous studies have demonstrated that the parent-adolescent discrepancies could be differentiated by parent gender, which might reflect fathers’ and mothers’ different parental roles (e.g., Ingoglia et al., 2021). Future studies should include a large sample composed of both fathers and mothers and an equal number of boys and girls to make investigations among four dyads, i.e., mother-boy, mother-girl, father-boy, and father-girl. Finally, the current study utilized a Chinese sample of parents and adolescents, which might demonstrate different patterns of use and perception of psychological control and autonomy support from the samples from other individualist contexts (e.g., Wang et al., 2007). Therefore, to obtain more comprehensive and generalizable understandings, cross-culture or multi-ethnicity design can be adopted in future studies. Nationally representative samples should be gathered to further strengthen the generalizability of findings.

## Conclusions

Parent-adolescent discrepant perceptions of parenting behaviors reflect family functioning and predict child outcomes. However, few studies have comprehensively examined the effects of parent-adolescent discrepancies in perceiving both psychological control and autonomy support on positive and negative aspects of adolescents’ adjustment, nor have they probed into the moderation by adolescent gender. Through latent difference score modeling, the current study showed that adolescents perceived higher psychological control and lower autonomy support than parents, and the psychological control discrepancies were larger for parent-boy dyads. The psychological control discrepancies predicted higher depression and autonomy support discrepancies predicted lower depression. The discrepancies in autonomy support were associated with boys’ depression rather than with girls’. The results suggested that parent-adolescent discrepancies in perceiving different parenting behaviors could contribute to adolescents’ adjustment through different processes and these processes might differ for boys and girls to some extent. These findings provided some practical implications that parents should appreciate adolescents’ needs for autonomy and adapt parenting strategies accordingly to facilitate adolescents’ psychological adjustment.

## Supplementary information

Online Resource
