# Supplementary material for: Parent-Adolescent Discrepancies in Perceiving Parental Psychological Control and Autonomy Support Predict Adolescents’ Psychological Adjustment: Does Adolescent Gender Make a Difference?
Source: J Youth Adolesc. 2025 Feb 1;54(6):1588–99. doi: 10.1007/s10964-025-02144-5 (PMC12137482; doi:10.1007/s10964-025-02144-5)
Supplement: Supplementary file 1 — Online Resource [file 10964_2025_2144_MOESM1_ESM.docx]

**Parent-Adolescent Discrepancies in Perceiving Parental Psychological Control and Autonomy Support Predict Adolescents’ Psychological Adjustment: Does Adolescent Gender Make a Difference?**

Online Resource

**Table S1**

*Descriptive Statistics and Zero-Order Correlations of Key Study Variables (Raw Scores)*

|  | 1 | 2 | 3 | 4 | 5 | 6 | 7 | 8 |
| --- | --- | --- | --- | --- | --- | --- | --- | --- |
| 1. Psychological control (parent) | – |  |  |  |  |  |  |  |
| 2. Psychological control (adolescent) | .26^***^ | – |  |  |  |  |  |  |
| 3. Autonomy support (parent) | .06 | –.06 | – |  |  |  |  |  |
| 4. Autonomy support (adolescent) | –.04 | –.09 | .24^***^ | – |  |  |  |  |
| 5. Depression (Time 1) | .12^*^ | .34^***^ | –.15^*^ | –.40^***^ | – |  |  |  |
| 6. Resilience (Time 1) | –.16^**^ | –.17^**^ | .09 | .26^**^ | –.46^***^ | – |  |  |
| 7. Depression (Time 2) | .10 | .24^***^ | –.16^**^ | –.33^***^ | .36^***^ | –.27^***^ | – |  |
| 8. Resilience (Time 2) | –.09 | –.11 | .12^*^ | .21^***^ | –.26^***^ | .43^***^ | –.45^***^ | – |
| *M* | 2.58 | 2.99 | 3.93 | 3.58 | 2.11 | 3.56 | 2.27 | 3.36 |
| *SD* | 0.97 | 1.17 | 0.70 | 1.09 | 0.96 | 0.84 | 1.07 | 0.71 |

*Note*. ^*^*p* < .05. ^**^*p* < .01. ^***^*p* < .001.

**Table S2**

*Confirmatory Factor Analysis: Model Fit Indices of Psychological Control and Autonomy Support*

|  | Adolescents’ reports | | | | | | Parents’ reports | | | | | |
| --- | --- | --- | --- | --- | --- | --- | --- | --- | --- | --- | --- | --- |
|  | χ^2^ | df | CFI | TLI | RMSEA | SRMR | χ^2^ | df | CFI | TLI | RMSEA | SRMR |
| Psychological control | 42.148 | 20 | .982 | .967 | .060 | .029 | 55.751 | 20 | .973 | .952 | .076 | .035 |
| Autonomy support | 53.893 | 18 | .968 | .951 | .080 | .024 | 48.548 | 18 | .963 | .943 | .074 | .033 |

*Note.* For psychological control, item 9 (“For things in my child’s life, I’m usually in charge”) in both parents’ and adolescents’ reports was deleted for low loadings (< .60). Residual variances of item 1 (“I let my child know that what I want him/her to do is the best for him/her and he/she should not question it”) and item 2 (“I tell my child of all the sacrifices I have made for him/her”), residual variances of item 1 and item 3 (“I tell my child that when he/she grows up, he/she will appreciate all the decisions I have made for him/her”), residual variances of item 2 and item 3, residual variances of item 3 and item 4 (“When I have an argument with my child, I say things like, ‘You’ll know better when you grow up’”); residual variances of item 3 and item 5 (“I let my child know that I am disappointed in him/her when he/she does not do things my way”); residual variances of item 5 and item 6 (“I let my child know that he/she should feel guilty when he/she does not meet my expectations of him/her”) were allowed to co-vary for both parents’ and adolescents’ reports. For autonomy support, residual variances of item 2 (“I listen to my child’s opinion or perspective when he/she has a problem”) and item 3 (“I allow my child to decide things for himself/herself”); residual variances of item 3 and item 6 (“I let me child make his/her own plans for things he/she wants to do”) were allowed to co-vary for both parents’ and adolescents’ reports. CFI = Comparative Fit Index; TLI = Tucker Lewis index; RMSEA = Root Mean Square Error of Approximation; SRMR = Standardized Root Mean Square Residual.

**Table S3**

*Multi-group Comparison for Boys and Girls (Psychological Control)*

|  |  | χ^2^ | df | CFI | RMSEA | Δχ^2^ | *p* |
| --- | --- | --- | --- | --- | --- | --- | --- |
| Model 0 |  | 694.094 | 473 | .946 | .055 |  |  |
| Model 1 | Time 1 Depression →Time 2 Depression | 696.672 | 474 | .946 | .055 | 2.578 | .108 |
| Model 2 | LDS mean → Time 2 Depression | 696.121 | 474 | .946 | .055 | 2.027 | .155 |
| Model 3 | Parent-report →Time 2 Depression | 694.080 | 474 | .947 | .055 | –0.014 | .906 |
| Model 4 | Time 1 Resilience → Time 2 Resilience | 696.161 | 474 | .946 | .055 | 2.067 | .151 |
| Model 5 | LDS mean → Time 2 Resilience | 696.038 | 474 | .946 | .055 | 1.944 | .163 |
| Model 6 | Parent-report → Time 2 Resilience | 695.600 | 474 | .946 | .055 | 1.506 | .220 |
| Model 7 | Fully Constrained | 708.588 | 479 | .944 | .056 | 14.494 | .025 |

*Note.* LDS = latent difference score; CFI = Comparative Fit Index; RMSEA = Root Mean Square Error of Approximation.

**Table S4**

*Multi-group Comparison for Boys and Girls (Autonomy Support)*

|  |  | χ^2^ | df | CFI | RMSEA | Δχ^2^ | *p* |
| --- | --- | --- | --- | --- | --- | --- | --- |
| Model 0 |  | 643.159 | 399 | .937 | .063 |  |  |
| Model 1 | Time 1 Depression → Time 2 Depression | 654.833 | 400 | .934 | .064 | 11.674 | <.001 |
| Model 2 | LDS mean → Time 2 Depression | 651.851 | 400 | .935 | .064 | 8.692 | .003 |
| Model 3 | Parent-report → Time 2 Depression | 645.500 | 400 | .936 | .063 | 2.341 | .126 |
| Model 4 | Time 1 Resilience → Time 2 Resilience | 644.289 | 400 | .937 | .063 | 1.130 | .288 |
| Model 5 | LDS mean → Time 2 Resilience | 643.517 | 400 | .937 | .063 | 0.358 | .550 |
| Model 6 | Parent-report → Time 2 Resilience | 644.024 | 400 | .937 | .063 | 0.865 | .352 |
| Model 7 | Fully Constrained | 658.553 | 405 | .944 | .056 | 15.394 | .017 |

*Note.* LDS = latent difference score; CFI = Comparative Fit Index; RMSEA = Root Mean Square Error of Approximation.

**Table S5**

*Sensitivity Analyses of the Associations between Parents’ Self-Reports, Parent-Child Difference Scores, and Adolescents’ Psychological Adjustment*

|  |  | Depression | | | Resilience | | |
| --- | --- | --- | --- | --- | --- | --- | --- |
|  |  | *B* | *SE* | *β* | *B* | *SE* | *β* |
| Parent self-report (psychological control) | Overall | 0.22 | 0.10 | **.16^*^** | –0.07 | 0.06 | –.08 |
| Latent difference score mean (psychological control) | Overall | 0.18 | 0.08 | **.19^*^** | –0.05 | 0.05 | –.08 |
| Parent self-report (autonomy support) | Overall | –0.33 | 0.10 | **–.20^**^** | 0.14 | 0.06 | **.13^*^** |
| Latent difference score mean (autonomy support) | Overall | –0.21 | 0.07 | **–.21^**^** | 0.06 | 0.04 | .08 |
| Latent difference score mean (autonomy support) | Boy | –0.34 | 0.08 | **–.37^***^** |  |  |  |
|  | Girl | –0.05 | 0.10 | –.05 |  |  |  |

*Note.* In sensitivity analyses, adolescents’ depression and resilience were entered into the models separately. Significant standardized coefficients are in bold. For multi-group analyses, only the path differing between boys and girls is presented. *B* = unstandardized estimate; *β* = standardized estimate; *SE* = standard errors.

^*^*p* < .05. ^**^*p* < .01. ^***^*p* < .001.
